# Fiber Patterns in Young Adults Living in Different Environments (USA, Spain, and Tunisia). Anthropometric and Lifestyle Characteristics

**DOI:** 10.3390/nu9091030

**Published:** 2017-09-18

**Authors:** María José García-Meseguer, Amalia Delicado-Soria, Ramón Serrano-Urrea

**Affiliations:** 1 Department of Nursing, Physiotherapy and Occupational Therapy, Faculty of Nursing, University of Castilla-La Mancha, Avda. de España, s/n, 02071 Albacete, Spain; amalia.delicado@gmail.com; 2 Department of Mathematics, Faculty of Computer Science Engineering, University of Castilla-La Mancha, Avda. de España, s/n, 02071 Albacete, Spain; ramon.serrano@uclm.es

**Keywords:** diet, fiber, socioeconomic factors, young adults, dietary pattern

## Abstract

Benefits of dietary fiber go beyond its effect on chronic diseases associated with development. Consequently, the pattern of fiber intake has been considered an indicator for diet quality. Young adults are especially vulnerable to a food environment that drives an increase in chronic diseases linked to economic development. The aim of this work was to characterize patterns of fiber intake among university students. A cross-sectional study was conducted on a sample of 730 students enrolled at the University of Castilla-La Mancha (Spain), the University of Carthage (Tunisia), and Florida International University (USA). Mean age was 21.2. Food consumption was self-reported in two 24-h recalls. Mean dietary fiber intake was 17.8 g, not reaching the adequate intake. Contrary to expectations, American participants were the highest consumers (*p* < 0.001), and also exhibited the highest BMI. Cereals, legumes, vegetables and fruit were the main food sources of fiber. Fiber from appetizers, prepared and precooked meals, sauces, spices and condiments accounted for 16.7% in American participants, 7.4% in Spanish participants and 2.6% in Tunisian participants. Total fiber intake increased with energy intake but did not depend on smoking habits and physical activity in any country. It is essential to improve consumers’ interpretation of guidelines on fiber intake.

## 1. Introduction

The association between fiber intake and health outcomes first came to public attention in the 1970s, due to the detection of diseases in Western countries not existing on the African continent [1,2] such as diverticulitis, constipation or hemorrhoids. In this regard, reduction of fiber intake is inherent in changes from traditional food patterns to Western food models. This has led to an important change in recommendations on food and health, with an increase in daily fiber intake being actively promoted [3,4,5]. The key role of fiber intake in intestinal health is now well established [6,7], and it is also known that fiber-rich dietary patterns [8] and an adequate fiber intake is related to prevention of chronic diseases such as type 2 diabetes, obesity or certain types of cancer, whose prevalence is rising as a result of economic development and globalization [9,10,11,12]. Anthropometric and lifestyle characteristics were recently shown to mediate the association between fiber-rich dietary patterns and risk of chronic disease [8]. All generations are being affected by a food environment that favors the prevalence of these diseases, but young adults are especially vulnerable because they usually lead independent lifestyles and are unaware of other types of food environment [13].

In this scenario, and in order to improve the health state of people from middle and high-income countries, World Health Organization (WHO) and Food Agriculture Organization (FAO) policy guidelines [14,15] include recommendations for fiber intake in countries’ dietary guidelines. Therefore, patterns of fiber consumption may be considered an indicator of diet quality [16].

Although there is no universally accepted definition of fiber [17,18,19], definitions from the Institute of Medicine [20] are those most commonly found in the literature. Thus, total fiber is considered as the sum of dietary fiber and functional fiber. Moreover, it is known that different types of fiber may have different effects on health [21,22,23,24]. Since there is no recommended dietary allowance (RDA) or estimated average requirement (EAR), Adequate Intake (AI) has been defined a recommended average daily total fiber consumption of 14 g/1000 Kcal for adults of both genders [20].

Fiber structure has long been an area of interest, and a relationship has been established between fermentability, which is linked to chemical properties, and satiety and laxation [25,26,27]. Moreover, other positive effects of dietary fiber on chronic diseases have also been identified. These benefits are related to the complex function of fiber as a carrier of phytochemicals and its effect on gut microbiota [28,29,30,31]. Recent studies evidence that further knowledge of fiber healthy properties requires including information about food sources [32,33,34].

Despite country guidelines showing different fiber food sources according to the characteristics of the local diet, other factors such as price, taste, convenience or trends can strongly influence food choice, which will ultimately affect daily total fiber intake. This is the case of young adults. This population group presents an important challenge due to the coincidence of a series of emotional, physiological and environmental changes. They are usually sensitive to the influence of trends, which includes the consumption of soft drinks, snacks, prepared and pre-cooked meals and other ready-to-eat products, most of which are rich in sugars and fats, and deficient in fiber [35,36,37]. The transition to an independent life can be very stressful and can influence food choice, especially if they are living away from their parents’ home and have poor cooking skills [38,39,40,41]. Epidemiological studies on this population group have shown that symptoms of diseases of affluence occur earlier than expected by biological age alone [42]. Thus, learning healthy food habits such as reaching an adequate fiber intake at an early age is critical for improving future health [43,44]. This is particularly important in university students, who are considered an important target group to promote healthy lifestyles [45].

To date, most studies have investigated food patterns of a country’s overall population and the relationships with different diseases. Research focusing on young adults is, however, still limited. Although some studies on fiber intake have been conducted in young adults living in countries with different food patterns and incomes [33,42,45,46,47,48], to the best of our knowledge, no study has simultaneously assessed diet quality by fiber patterns in young people with homogeneous demographic and education characteristics but living in different environments (social, geographic, cultural, and economic). Knowledge of food patterns and fiber intake in young people from different countries, and the degree to which recommendations are followed, can help understand the complex relationships between young adults’ behavior and food in different contexts.

Tunisia, Spain and the USA have different levels of development and different socio-cultural characteristics that directly influence their food patterns. In Tunisia, a low-middle income country [49], fiber consumption is decreasing mainly in urban areas, while the prevalence of chronic diseases inherent in globalization is rising [50,51]. Spain, a country where the Mediterranean food pattern is becoming less common, also presents lower total fiber consumption than recommendations [52]. In the United States, a Western model, guidelines promote strategies to increase fiber intake to improve American people’s health [53,54]. Recent research in these countries has shown a decrease in fiber consumption among young adults relative to adult and older adult age groups [55,56,57].

The aim of this work was to characterize fiber consumption patterns of students at Florida International University (USA), the University of Castilla-La Mancha (Spain) and the University of Carthage (Tunisia), and to identify potential determinant factors.

## 2. Materials and Methods

### 2.1. Study Design

A cross-sectional survey was administered to students enrolled at the University of Castilla-La Mancha, campus of Albacete, Spain (hereafter UCLM), the University of Carthage, Tunisian Republic (hereafter UCA) and Florida International University, Miami, FL, USA (hereafter FIU) during 2013. All procedures were in accordance with the WMA Declaration of Helsinki (Ethical Principles for Medical Research Involving Human Subjects). Informed consent was provided by each participant. The projects were approved by the following Ethics Committees: “Food habits of college students: University of Castilla-La Mancha (Albacete Campus)”, approved by the Ethics Committee at the University Hospital Complex of Albacete (CEIC), Act No. 02/13, January 29, 2013; “Food habits of college students: Florida International University”, approved by CEIC, Act No. 10/13, October 28, 2013, and FIU Institutional Review Board, IRB-13-0231, June 26, 2013; and “Food habits in a university population: Tunis Virtual University and the University of November 7 at Carthage (Tunisia)” approved by CEIC, Act No. 11/13, November 25, 2013.

### 2.2. Study Participants

Subjects were recruited using stratified sampling according to students enrolled on each degree course at the three campuses. Inclusion criteria were: (1) being enrolled at one of the three universities during 2013; (2) being aged between 18 and 30 years; (3) voluntarily agreeing to participate in the erratumsurvey; and (4) accepting and signing the informed consent. Exclusion criteria were: (1) not completing all the data in the surveys; and (2) presence of acute diseases (affecting diet) when the surveys were administered. In addition, an exclusion limit criterion was established, following the recommended intakes: (3) males whose daily energy intake was higher than 4000 Kcal/day and less than 800 Kcal/day, and females whose daily energy intake was higher than 3500 Kcal/day and less than 500 Kcal/day [58].

### 2.3. General Data

General information was self-reported by each subject using a questionnaire including the following items: (1) demographic data: gender, age; (2) anthropometric measurements: weight, height; (3) weight-loss diet: yes/no and (4) smoking habits: non smoker, ≤5 cigarettes/day and >5 cigarettes/day. Body mass index (BMI) (Kg/m^2^) was calculated from anthropometric data and individuals were classified into four categories: underweight (BMI < 18.5), normal range (18.5 ≤ BMI ≤ 24.9), overweight (25 ≤ BMI ≤ 29.9) and obese (BMI ≥ 30) [59].

### 2.4. Food Consumption Assessment

Food consumption data was gathered by two non-consecutive 24-h recalls including one weekend day. All food and beverages consumed were recorded by the recruited individuals. Trained dietary nurses explained how to complete the questionnaires, administered them and checked the data recorded. To help estimate portion sizes, participants were shown images of household measures and a Visual Guide [60,61].

Dial program 3.3.5 (Alceingenieria, Madrid, Spain) was used to determine energy and nutrients intake. Physical Activity Level (PAL) was calculated as the ratio of total to basal daily energy expenditure and classified individuals as: sedentary (1.0 ≤ PAL < 1.4), low active (1.4 ≤ PAL < 1.6), active (1.6 ≤ PAL < 1.9) and very active (1.9 ≤ PAL < 2.5) [62].

Daily fiber intake was calculated for each subject. The Dial program predetermines the following food group classification: cereals, legumes, vegetables, fruits and nuts, dairy products, meat, fish, eggs, sugar, sweets and pastries, fat and oil, non-dairy beverages, prepared and precooked meals, snacks, sauces, spices and condiments. Across these groups, all the foods that contributed to total fiber intake were reflected.

The assessment was carried out using the following references: Tables and Dietary References of the Institute of Medicine [20], Tunisian and Spanish Food Composition Tables [63,64], and USDA Nutrient Database [65].

Taking into account the AI (14 g/1000 Kcal for both genders [20]), various approaches have been utilized in order to evaluate or define a level for a diagnosis of risk of insufficient fiber intake. Of these, a value of two thirds of AI has been accepted in different studies [66,67]. In our study, we calculated the percentage of students consuming less than two thirds of AI, those whose intakes were more than two thirds of AI but less than AI, and those who reached AI.

### 2.5. Statistical Analysis

Statistical analysis was performed with IBM SPSS 22 (SPSS Inc., Chicago, IL, USA). Medians (and interquartile ranges) and means (and standard deviations) were used as descriptive statistics for quantitative variables. Proportions were used to describe qualitative variables. Kolmogorov-Smirnov and Shapiro-Wilk tests were used to study the normality of the distributions. The student’s *t* test (with previous Levene’s test for equality of variances) and the Mann-Whitney U test were used to compare two independent samples. The Kruskal-Wallis test (and Dunn post hoc method) were performed to compare more than two independent samples. When appropriate, trend analysis was performed using the Jonckheere-Terpstra trend test. The Chi-square test (*χ*^2^) and the likelihood ratio test were used to compare proportions. Correlations were evaluated according to the Spearman’s correlation coefficient. Level of significance was established as a *p*-value < 0.05.

## 3. Results

A total of 775 students meeting inclusion criteria completed the surveys, providing all the required information. Nine were excluded due to the presence of diseases and 36 more individuals were excluded, as they did not meet daily energy intake limits. Therefore, the final sample consisted of 730 students (272 students from UCLM, 132 from UCA and 326 from FIU).

Mean age was 21.2 years, and gender distribution was 491 females and 239 males. It was a non-obese population (only 6.3% of the students showed obesity), whose mean BMI was 22.9 Kg/m^2^. Most of the students reported they were not smokers (88.1%) and followed no weight-loss diets (86.0%). Regarding the level of physical activity, 63.0% of the students were found to be active or very active. Mean daily energy intake was around 2000 Kcal and mean daily total fiber intake was around 18 g. We found statistically significant differences between females and males: energy intake was higher in males, and the percentages of overweight and obese students, as well as the percentage of smokers, were also higher in males (Table 1). BMI, total fiber intake and energy intake differences between females and males are shown in Table 2. It is interesting to note that only one fifth of the students at FIU classified as overweight or obese were following a diet when surveyed. Gender distribution showed that the percentage of American male students whose BMI was above the normal range was significantly higher than the percentage found in females (47.9% vs. 25.7%). Complete information about sociodemographic, anthropometric and lifestyle characteristics by country and gender are reported in Tables S1–S3 in supplementary material.

Cross-country analysis showed different lifestyle habits (Table 3). Most of the variables studied showed significant differences between the students from the three universities: total energy intake, total fiber intake, tobacco consumption, physical activity, and weight-loss diets. The BMI variable also showed significant differences between the universities. Our results showed that the percentage of students at UCLM classified as overweight or obese (18.4%) was more than twice the percentage of overweight or obese students at UCA (9.1%). This percentage was even higher in American participants (30.6%), being more than three times higher than that of Tunisian students.

Physical activity also has an important role in a healthy lifestyle. Although the overall sample showed a population consisting mainly of active or very active students, significant differences were found when the three countries were studied separately. While the percentage of sedentary or low active Spanish students reached almost 70%, this percentage was only 28% in Tunisian students and barely 13.5% in the American sample.

Regarding daily fiber consumption, Table 3 shows significant differences between the students from FIU and students from UCA and UCLM. American participants presented the highest intakes, reaching mean and median values of around 20 g/day, while these values were only around 15 g/day in students from Mediterranean countries. As expected, according to the results obtained for total daily fiber intake, we found significant differences for fiber intake adjusted by energy between Mediterranean and American participants.

Table 4 shows daily total fiber intake distribution among the different categories for those variables presenting significant comparisons. Information on all the variables is shown in Tables S4–S6 in supplementary material. For each country, no statistically significant differences in fiber intake were found when smoking habits and level of physical activity variables were studied. Significant differences by gender were found in students from UCLM: male students showed higher daily total fiber intake than females. In Spain and Tunisia, no differences in fiber intake were found between students following and not following weight-loss diets. However, results were different among American participants: students not following weight-loss diets consumed less fiber than those who were (a difference of almost 7 g/day on average). Regardless of gender, a significantly positive association between energy intake and fiber intake was found in the three countries.

Although when the BMI variable was categorized, no significant differences in fiber intake was found among the four categories, a negative correlation between total fiber intake and BMI was found in students from FIU when BMI was considered as a continuous variable. Further analysis by gender showed a negative association between these variables in females but no association was found in males (Table 4).

**Figure 1:**
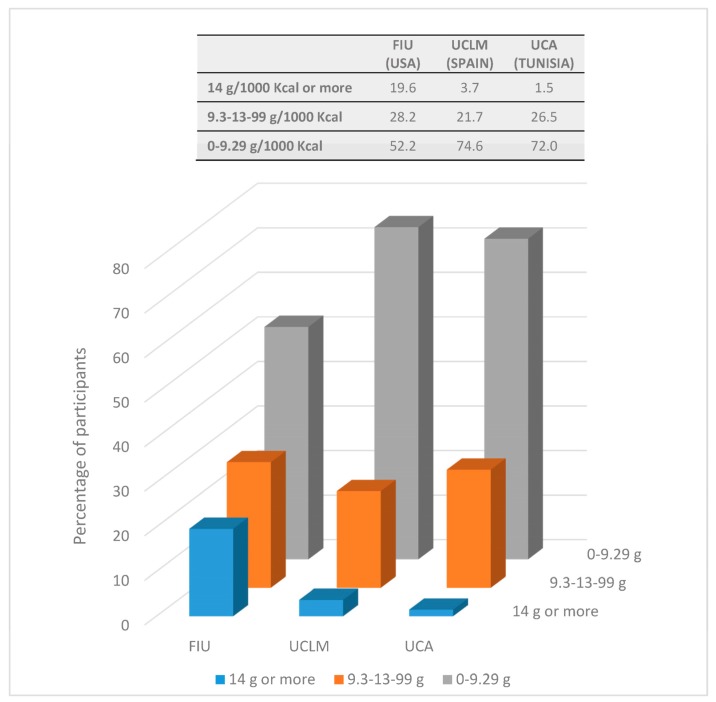
Percentage of participants consuming less than two thirds of the AI, those who consumed between two thirds of the AI and the AI, and those consuming at least the AI (14 g/1000 Kcal). AI: Adequate Intake; FIU: Florida International University; UCLM: University of Castilla-La Mancha; UCA: University of Carthage.

Table 5 shows fiber intake (g/1000 Kcal) by the food groups providing the largest amounts of fiber (cereals, legumes, vegetables and fruits) for all students from UCLM, FIU and UCA. In all cases, mean total fiber intake by country did not reach AI. A large percentage of Tunisian/Spanish (>96%) and American (>80%) subjects did not reach adequate fiber intake. Moreover, around 70% of Mediterranean students and more than 50% of American students did not even reach two thirds (9.3 g/1000 kcal) of AI (Figure 1).

The study of the individuals not reaching two thirds of AI showed significant differences between Tunisian and Spanish/American students: subjects from UCA consumed about 0.6 g/1000 Kcal more than the students from UCLM or FIU (Table 5).

Regarding the origin of the fiber, our results showed that 76.6% of the fiber came from cereals, legumes, vegetables and fruits (including nuts). This percentage reached 82.6% in students from UCA. In all countries, cereals provided an average of at least 3 g/1000 Kcal. The sum of vegetables and fruits provided 3.7 g/1000 Kcal of fiber in students from FIU and 2.8/2.6 g/1000 Kcal in Tunisian/Spanish participants, respectively. It is interesting to note that fiber consumption from legumes reported by American students was more than twice that reported by Mediterranean students. In the three countries, cereals were the main source of fiber for people with fiber intake below AI. Regarding individuals reaching AI, cereals were the major source of fiber among Spanish students while the main source of fiber among American and Tunisian students was fruit.

**Figure 2:**
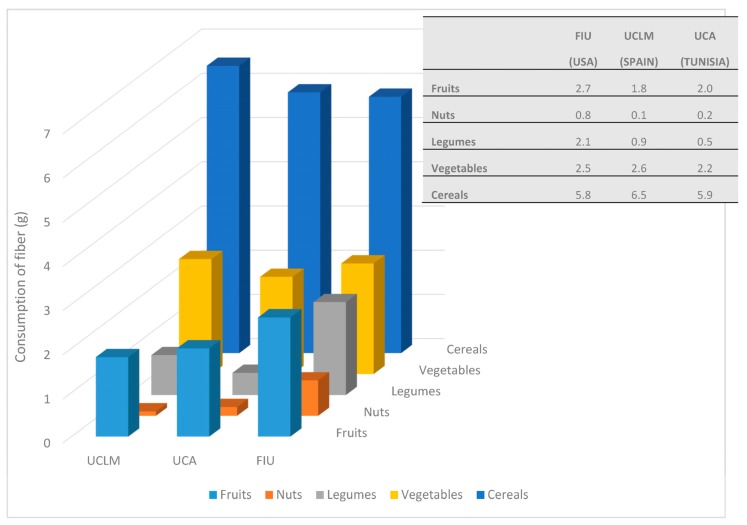
Consumption of fiber (g) from the main food groups that contribute to total fiber intake.

Consumption of fiber (g) from the main groups that contribute to total fiber intake is shown in Figure 2. The distribution of the consumption of high-fiber food groups by quartiles of total fiber intake is shown in Table 6. For each country, total fiber intake was significantly different when quartiles were compared. However, participants from different countries showed some differences in the main food groups that contributed to increase total fiber intake. Given that recent investigations have shown that nuts play an important role in health [68], Table 6 and Figure 2 show specific additional information about fiber intake from nuts.

In line with previous results, when fiber intake per thousand kilocalories was studied (Table 5), the results in Table 6 also show that in our population the main source of total fiber was cereals, followed by vegetables and fruits. For each country (Spain, Tunisia and the USA), the amount of total fiber obtained from cereals (6.5, 5.9 and 5.8 g on average, respectively) was two to three times higher than the amount of fiber from the groups that ranked second: vegetables (2.6, 2.2 and 2.5 g on average, respectively) and fruits (1.8, 2.0 and 2.7 g on average, respectively).

It is interesting to note that, in students from UCA, the increased consumption of fiber from the first to the fourth quartiles came from fruit consumption (fiber intake increases 2.8 g on average), followed by vegetables and cereals (fiber intake increases around 2 g on average in each group). In relative terms, fiber obtained from fruits increased five-fold between the first and the last quartiles while fiber from vegetables and cereals increased 2.2 and 1.4 times, respectively. In relative terms, fiber obtained from legumes and nuts increased ten-fold between the first and the last quartiles. These intakes increased by nearly 1 g and 0.5 g, respectively, in absolute terms.

In students from FIU, more food groups contributed to the increase in total fiber from the first to the fourth quartiles. In the legume group, mean fiber intake in the first quartile was 0.3, while mean fiber intake in the last quartile was 6.3, which is an increase of 6 g (in relative terms, this fiber from legumes increased around 20 times). Mean fiber consumption from fruits increased from 1.0 g to 4.3 g, mean fiber consumption from vegetables increased from 1.7 g to 3.1 g, and mean fiber consumption from cereals increased from 4.6 g to 6.4 g. In relative terms, fiber from these groups increased 4.3, 1.8 and 1.4 times, respectively. Finally, in relative terms, fiber obtained from nuts increased 21-fold between the first and the last quartiles. This intake was an increase of nearly 1 g in absolute terms.

Among participants from UCLM, the increased consumption of fiber from the first to the fourth quartiles came from cereal consumption (in absolute terms, an increase of almost 4 g between the first and the fourth quartiles was found), followed by fruits, legumes and vegetables (differences of 2.6, 2.4 and 1.4 g, respectively, were observed). In relative terms, in line with the results for American participants, the most important increase was found in the legume group (fiber obtained from legumes increased 13-fold between the first and the last quartiles), followed by the fruit group (fiber from fruits increased 4.3 times) and vegetable and cereal groups (fiber from these groups increased 1.8 times). It is interesting to highlight that fiber from nuts remained practically constant in all quartiles.

**Figure 3:**
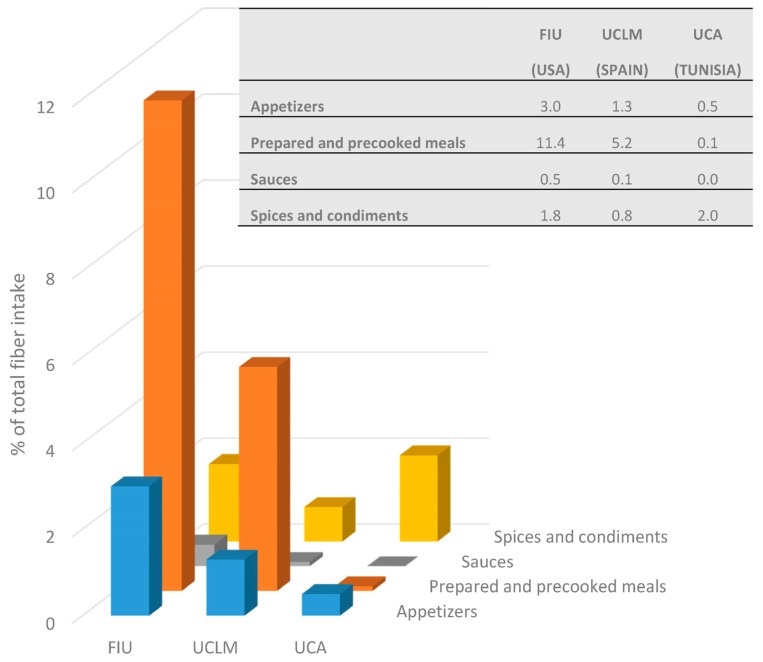
Fiber from appetizers, prepared and precooked meals, sauces, and spices and condiments (% of total fiber intake).

Although cereals, legumes, vegetables and fruit are the main fiber sources, young people usually consume pre-cooked foodstuff or ready-to-eat meals. Figure 3 shows fiber intake from appetizers, ready-to-eat meals, and sauces and condiments. The last group includes both healthy aromatic herbs, spices and seasonings, and complex sauces. In supplementary material Table S7 shows detailed information about these groups. Our results showed that even though intake for the main fiber food groups was highly homogenous across participants, differences appear when these other groups are studied. A total of 16.7% of fiber consumed by American participants was obtained from appetizers, prepared and precooked meals, and sauces and spices and condiments groups. This percentage was relatively small in Mediterranean participants. Spanish participants only reached 7.4% and Tunisian participants barely reached 2.6%. It is worth highlighting the virtually non-existent fiber intake from both prepared and precooked meals and sauces among Tunisian participants. If we omit the spices and condiments group, fiber intake from prepared and precooked products by students from FIU is almost 25 times higher than among students from UCA and double that of students from UCLM.

## 4. Discussion

Economic and industrial development has led to changes in demographic structures, health and food patterns. New models of transitional nutrition characterized by loss of food culture are emerging. Decreased fiber intake is one of the characteristics of these new models traditionally linked to new health risks [69,70]. Benefits of dietary fiber intake go beyond effects on chronic diseases associated with developed economies (type 2 diabetes, cardiovascular diseases, cancer and others) [9,10], besides the well-known impact on gastrointestinal diseases [1,2,25]. Recent research also suggests positive effects on other illnesses and conditions (infections or respiratory diseases) and negative effects on all the total death rates [12,71]. In this context, the pattern of fiber intake is considered an indicator of diet quality [16,27] and international policy guidelines [14,15] have included the intake of food sources with high fiber content in order to meet the recommendations.

Although qualitative differences between fiber food sources have been found according to availability, cultural and social characteristics, taboos and the economic development of countries, young adults are highly sensitive to trends, ready-to-eat meals being an example. Factors such as leaving school, going to university, leaving the parental home and having no cooking skills lead young people to be more affected by an unhealthy food environment [13,41]. The fiber pattern intake will persist for a lifetime in many cases [72]. Currently, there is great scientific interest in research on fiber and its food sources, as some long-term benefits of the former may depend on the latter [73,74]. Therefore, the pattern of fiber intake in young adults, like other nutrients, plays a key role in improving future health state.

The present study on diet quality measured by fiber patterns in young adults was carried out with data from students enrolled at the University of Castilla-La Mancha (Spain), the University of Carthage (Tunisia) and Florida International University (USA). There is extensive research on food patterns in university students around the world. Some works record fiber intake, in addition to other nutritive diet features [38,39,40,42,75], while others study the effect of fiber intake on certain diseases [12,76,77] or investigate food sources of fiber in a specific country [33,56,57,78]. However, to the best of our knowledge, none of them has specifically addressed fiber consumption patterns in young adults with a high level of education but with different cultures and incomes, living in three countries with different levels of development and located on three continents. Tunisia is a developing country currently classified as low-middle income (but classified as middle-income when data were collected) [48], where traditional Mediterranean culture is supplemented by some French influences from recent centuries. Spain is another Mediterranean country, classified as high-income, and the USA is considered the prototype of the Western food model. Specific issues characterize the region where each University is located. The UCLM is located in a Spanish area in economic transition within the European Union [79]. A high percentage of students come from the same region. Miami, where FIU is situated, is inhabited by a large proportion of Latin Americans together other ethnic and cultural communities. UCA is located in the capital of Tunisia, one of the largest urban areas in the country [80].

The relationship between economic development and type of food consumed, with different consequences for health, generates models of transitional nutrition characterized by loss of both cultural and healthy food habits [69]. The impact in middle- and high-income areas leads to the adoption of unhealthier lifestyles and a shift away from healthier traditional ones. Thus, transition from the Mediterranean food pattern to the Western model implies a reduction in fiber intake and increased overall intake of saturated fat and sugar from precooked meals, snacks and other ready-to- eat meals. Regarding the age range of the population studied, young adults are the most susceptible to advertising, cultural trends and attitudes, so rapid changes in food behavior towards new tendencies are common [37,81]. Moreover, the positive relationship between level of education and healthy food habits is also well established [82]. The large number of potential sources of interpersonal variation that emerge when analyzing a population homogenous in aspects such as level of education and age, but different in cultural features and economic development [58], may provide an excellent opportunity to deepen knowledge of the complex relationship between health and diet. Our work addresses the study of patterns of fiber intake in young adults through analysis of their food sources, and discusses the impact of culture and economic development of the countries and other factors on these patterns.

According to their pattern of fiber intake, the diet quality of the population studied is low, since the participants did not reach the AI, in line with other populations of similar characteristics [42,57,75]. However, cultural and economic factors affect patterns of fiber intake. Contrary to expectations, students from FIU consumed more fiber than students from UCA and UCLM, countries that share Mediterranean traditional culture and have lower incomes than USA. Our results also revealed that total fiber intake increased with energy intake but did not depend on smoking habits and physical activity in any country. Moreover, only among the USA participants did the following of a weight-loss diet influence the pattern of fiber intake.

Regardless of country, the main food groups contributing fiber to the diet were cereals, legumes, vegetables and fruits. The distribution of the main sources of fiber consumed by our students was different in each country. The cereal group provided the largest amount of fiber in the three countries, but while vegetables were the second group providing fiber among Spanish and Tunisian students, the fruit group was the second group among American students.

Generally, analyses conducted at the levels of nutrients, food groups and possible overall dietary patterns yield the most complete information about diet [58]. Therefore, these three levels of study have been detailed in the following discussion. In the first level, fiber as a nutrient has been considered and data about fiber intake have been analyzed. The second level comprises the study of food groups providing fiber and, in the third level, the analysis of dietary patterns is addressed.

### 4.1. First Level

Our students showed low levels of fiber intake. Only one out of ten participants consumed an adequate amount of fiber, as defined by recommendations. The mean intake of participants did not reach the recommended levels (AI higher or equal to 14 g/1000 Kcal), which are based on scientific evidence of beneficial effects of fiber on chronic degenerative diseases [20]. At first sight, fiber intake runs parallel to the trend towards lower consumption of fiber associated with economic development of countries [81]. Thus, nutritional deficiencies associated with poverty are resolved, but, simultaneously, this environment involves harmful effects on health. Taking into account the level of education [82] , our results are in line with data on mean fiber intake found in other studies performed with young adults in these countries [56,57,75,83] and also with university students from other European countries such as Poland [42] and Greece [84]. However, in our survey total fiber intake was lower than levels of consumption reported in other European countries among adult population [85].

The results for mean fiber intake found in each of the three countries studied were significantly different and, contrary to expectations, showed that American participants were the largest consumers of fiber. The study of the range of variation between the students with the lowest and the highest levels of fiber intake showed different results in UCA when compared with UCLM and FIU. In this sense, Tunisian participants showed a lower range of variation than Spanish and American participants, with the Americans exhibiting the widest range of variation. Differences in income among the countries could be one of the influencing factors.

Our study demonstrates the impact of a country’s economic development on BMI, since one third of the American participants recorded excess weight, compared with one of ten of the Tunisians, with the Spanish students occupying the intermediate position. Only 5% of American participants followed weight-loss diets. In American students fiber consumption also decreased when body weight increased and only overweight and obese females showed a negative association between fiber intake and BMI. This gender-related difference coincides with other previous studies in young and middle-aged US adults [35], where a low-fiber and high-fat diet was associated with the greatest risk of excess weight in women. Studies among obese Tunisian females reveal similar results [86]. This association was not found in Spanish, Tunisian, and non-overweight American participants, which is in line with results from other surveys conducted in Japanese or Swedish populations [77,87]. In young and adult populations from Northern European countries (Germany, Denmark and the UK), a negative association between fiber intake and weight has also been found, but other studies performed in Mediterranean countries such as Italy reported no association between weight and fiber intake [85]. These differences could be explained, at least in part, by the low proportion of people classified as overweight or obese among our Mediterranean participants, as also happens among Italian participants [85] and Swedish and Japanese populations [77,87].

A negative correlation between BMI and fiber intake was found in each country. However, USA students showed the highest mean BMI and the highest mean fiber intake, in spite of the Western-diet consumption. Most probably, differences in food sources derived from the economic development may account for the paradoxical clue.

As is often the case with most nutrients in free-living populations, fiber intake increased with energy intake in our students, independently of country and gender, although other studies have found a negative correlation between fiber intake and energy intake in females [88].

### 4.2. Second Level

Incomplete understanding of the effects of healthy fiber has guided research towards the investigation of food sources of fiber. In this section, we have discussed the main food groups contributing to total dietary fiber intake (cereals, legumes, vegetables and fruit). In addition, other minority groups were studied (appetizers, ready-to-eat meals, and sauces and condiments), due to possible different effects on health resulting from their consumption [36,37,89]. Regarding the main food groups, and according to current knowledge, different health benefits of fiber depend on criteria of viscosity and fermentability. Less fermentable fibers increase fecal weight to a greater degree than more fermentable fibers. Thus, the effect on fecal weight of fiber from cereals and vegetables is similar, and is higher than the effect of fiber from fruits [32]. Moreover, high consumption of fruits or fiber-fruit has been found to decrease the risk of cardiovascular disease in young Mediterranean people [33]. In our study, regardless of the country, the cereal group provided the highest amount of consumed fiber. It is interesting to highlight the high levels of fiber from legumes (string beans) consumed by our American students, which is in line with other studies in child and adult populations [16]. These outcomes matched those found in different surveys in the same countries and carried out among young and/or adult population when main food groups were studied [12,57,75]. However, when we considered the individuals reaching the recommended fiber intake, Tunisian participants showed the highest levels of fiber intake from fruits. This might provide greater protection against cardiovascular diseases in these young Mediterranean students.

Controversy in some results and the incidence of related chronic diseases has led researchers to suggest that quantity of fiber and the effect of its chemical properties on health are insufficient to fully understand its healthy effects. In this sense, the importance of fiber in the complex matrix of relationships between nutrients lies in its capacity to facilitate interaction among food components [27,30].

Regarding the minor food groups that contribute to total fiber intake, studies on food consumption commonly include as sources of fiber and nutrients groups like appetizers, ready-to-eat meals, and sauces and condiments [39,56,57]. These food groups are usually consumed by the studied population, arguably due to reasons such as trends, price or lack of time for cooking. The relationship between numerous foods belonging to these groups and chronic diseases linked to economic development such as gastrointestinal diseases, obesity and type 2 diabetes, among others, is well established [13,90]. Fiber-enriched snacks also often contain a higher percentage of energy from carbohydrates and added sugars [36]. Thus, promoting improved patterns of fiber intake regardless of the fiber source could have counterproductive effects [89,90].

While considering the food groups that most contribute to fiber intake (legumes, cereals, fruit and vegetables) the students’ behavior is similar, when we analyze the groups that contribute less fiber, significant differences appear. The high fiber intake from these products among the American students contrasts with the absence of intake from sauces and precooked meals among their Tunisian counterparts. Furthermore, the idea that the beneficial properties of fiber may be isolated from intrinsically healthy foods, and added as ingredients to other foods perceived to be less healthy, is not always feasible, since the matrix of intact fiber may be affected during processing and, consequently, its healthy properties could be modified [27].

It should also be taken into account that even canned food with high levels of fiber such as legumes contain unhealthy ingredients not used in culinary preparation [16,91]. This could be the case of the students from FIU. Consumption of canned beans showed a major impact on fiber intake differences between the lowest and the highest American fiber consumers. In Spanish students, this increase was mainly due to the differences in fiber consumption from cereals. In contrast, differences between the lowest and the highest Tunisian fiber consumers were mainly accounted for by the differences in fiber consumption in the fruit group, it being important to note that most fruit consumed in Tunisia is natural whole fruit.

### 4.3. Third Level

The pattern of fiber intake among young adults with a high level of education reflects the impact of globalization and cultural factors in their countries of origin, both in the amount of fiber consumed and the main food sources of fiber. Low levels of fiber intake were observed and none of the countries reached recommended levels. However, high-income countries are more aware of the need to increase fiber intake in order to improve health. In these countries, a large variety of cheap and easy to eat fiber-enriched food, much appreciated by young adults, is available (snacks, bran breakfast cereals) [36,92]. This fact could clearly contribute to enhanced fiber intake. Furthermore, when the groups of appetizers, ready-to-eat meals, and sauces were analyzed, the large differences found between participants reflect the influence of countries’ different food environments even in a population as homogeneous as young adults with a high level of education.

Among chronic diseases associated with economic development, obesity is one of the most serious public health problems [70], and fiber is associated with satiety [26,27]. Therefore, the relationship between fiber intake and excess weight is of great interest [34,55,85], especially since we know that younger adults are now gaining weight at a faster rate than the generation of their parents [93]. In this sense, our work confirms the effect of economic development on BMI since the prevalence of people with a BMI above the normal range (obese and overweight) was significantly lower in Tunisian participants than in American ones.

Taking into account the increase of pandemic chronic diseases and deaths caused by cancer and the difficulty of finding biomarkers for fiber intake [94,95], this study could provide a useful opportunity to reflect on the consequences of economic development and an incorrect interpretation of guidelines [96]. In line with other studies [19,27], this survey stresses the need to redefine the concept of fiber, and to further study the health effects of fiber intake from different food sources. Therefore, more studies are needed in different populations, including young adults, given the importance for the future of improving habits during this life stage.

Finally, it should be noted that a strength of this study is the homogeneity in the data collection process. A small, qualified team collected data in the three countries. The entire process was developed in consultation and coordination with experts from each University, who also advised on specific issues in each country. The high educational level of our population guarantees the quality and validity of the self-reported information. However, some limitations of this study are those inherent to the use of 24-h recalls. Omission of some food intakes (such as snacks or side-dishes) and inaccuracy of estimation of portion size are potential sources of error when 24 h recalls are used. Moreover, other factors such as season may contribute to daily variation in nutrient intakes which is largely depending on cultural and food availability. In low-middle income countries seasonal effects are relatively strong. This could be the case of Tunisia where data were collected in winter, so fruit and vegetables rich in fiber are more scarce. Therefore actual fiber consumption could be higher than results obtained in this analysis. In the USA and Spain, with extensive food preservation and transportation systems, seasonal effects are relative weaker.

On the other hand, in each country, students from a specific university are going to have a different cultural and ethnic background than in other parts of the country. Therefore, their dietary intake could differ from students in other areas of the country or the general population.

## 5. Conclusions

In this work, none of the countries studied reached recommended levels of fiber intake. According to both the current definition of dietary fiber and the established adequate intakes, the conclusion of this work is that the diet quality determined by fiber intake of students from FIU is higher than that of Mediterranean students (UCA and UCLM). This finding, however, does not coincide with the classic hypothesis about the impact of economic development on fiber intake: “economic development is linked to a decrease in fiber intake”. Total fiber intake increased with energy intake but did not depend on smoking habits and physical activity in any country.

Regardless of country, the main food groups that contributed fiber to the diet were cereals, legumes, vegetables and fruits. The cereal group provided the largest amount of fiber in the three countries, but while vegetables were the second group providing fiber among Spanish and Tunisian participants, fruit was the second group among American participants.

Although fiber intake showed great homogeneity among participants regarding the main fiber food groups, differences were found when appetizers, ready-to-eat meals, and sauces and condiments were analyzed. Students from FIU obtained almost a fifth of their total fiber intake from these groups, which is twice the percentage for students from UCLM and seven times that among students from UCA.

The conclusions of this work apply to the Universities studied in each country. However, the limitations of the study do not let us extrapolate the results to the countries as a whole.

## Figures and Tables

**Table 1:** Sociodemographic, anthropometric and lifestyle characteristics of the subjects (total sample).

	Total Sample (*n* = 730)	Men (*n* = 239)	Women (*n* = 491)	*p*
Population (%)	100	32.7	67.3	-
Age (years)				*p* = 0.705 ^†^
Mean + SD (95% CI)	21.2 ± 2.8 (21.0–21.4)	21.2 ± 2.9 (20.9–21.6)	21.2 ± 2.7 (21.0–21.5)	
Median (IR)	21 (4)	20 (4)	21 (3)	
Weight (Kg)				*p* < 0.001 ^†,^*
Mean + SD (95% CI)	65.0 ± 13.3 (64.0–65.9)	75.7 ± 12.3 (74.1–77.2)	59.8 ± 10.3 (58.9–60.7)	
Median (IR)	63 (17.0)	74.0 (16.0)	59.0 (12.0)	
BMI (Kg/m^2^)				*p* < 0.001 ^†,^*
Mean + SD (95% CI)	22.9 ± 13.3 (22.6–23.2)	23.7 ± 4.1 (23.2–24.3)	22.4 ± 4.0 (22.1–22.8)	
Median (IR)	22.3 (4.4)	23.3 (4.3)	21.8 (4.2)	
BMI-categories (%)				
Underweight	8.2	6.7	9.0	*p* = 0.011 ^§,^*
Normal range	69.6	64.4	72.1	
Overweight	15.9	19.2	14.3	
Obese	6.3	9.6	4.7	
BMI-2-categories (%)				
Underw. + normal rg.	77.8	71.1	81.1	*p* = 0.002 ^§,^*
Overweight + obese	22.2	28.9	18.9	
Total fiber intake (g)				
Mean + SD (95% CI)	17.8 ± 9.6 (17.1–18.5)	17.2 ± 8.4 (16.2–18.3)	18.1 ± 10.1 (17.2–19.0)	*p* = 0.303 ^†^
Median (IR)	15.6 (8.6)	14.9 (8.3)	15.7 (9.0)	
Energy intake (Kcal/day)				
Mean + SD	1971.9 ± 553.0	2070.2 ± 604.9	1924.1 ± 519.9	*p* = 0.001 ^‡,^*
(95% CI)	(1931.8–2012.1)	(1993.1–2147.3)	(1878.0–1970.2)	
Median (IR)	1932.5 (747.9)	2041.0 (897.6)	1889.0 (730.0)	
Weight-loss diet (%)	14.0	14.6	13.6	*p* = 0.715 ^§^
Smoking habits (%)				
Non-smoker	88.1	83.7	90.2	*p* = 0.029 ^§,^*
≤5 cigarettes per day	6.3	7.9	5.5	
>5 cigarettes per day	5.6	8.4	4.3	
Physical activity (%)				
Sedentary	9.0	7.5	9.8	*p* < 0.001 ^§,^*
Low active	27.9	28.5	27.7	
Active	40.3	32.6	44.0	
Very active	22.7	31.4	18.5	

SD: Standard deviation; CI: Confidence interval; IR: Interquartile range; BMI: Body mass index. ^†^ Mann-Whitney U test; ^‡^ Student’s *t* test; ^§^ *χ*^2^ test; * Significant differences.

**Table 2:** Body mass index (BMI), fiber intake and energy intake characteristics of the sample by universities.

**UCLM (SPAIN)**				
	**Total Sample (*n* = 272)**	**Men (*n* = 120)**	**Women (*n* = 152)**	***p***
Population (%)	100	44.12	55.88	-
BMI (Kg/m^2^)				*p* = 0.041 ^†,^*
Mean + SD (95% CI)	22.4 ± 3.3 (22.0–22.8)	22.8 ± 3.4 (22.2–23.4)	22.1 ± 3.2 (21.5–22.6)	
Median (IR)	22.0 (4.1)	22.7 (4.2)	21.6 (3.8)	
BMI-categories (%)				
Underweight	8.5	10	7.2	*p* = 0.297 ^§§^
Normal range	73.2	67.5	77.7	
Overweight	14.7	17.5	12.5	
Obese	3.7	5.0	2.6	
BMI-2-categories (%)				*p* = 0.119 ^§^
Underw. + normal rg.	81.7	77.5	84.9	
Overweight + obese	18.4	22.5	15.1	
Total fiber intake (g)				
Mean + SD (95% CI)	15.7 ± 6.6 (14.9–16.5)	16.4 ± 6.3 (15.3–17.5)	15.1 ± 6.9 (14.0–16.2)	*p* = 0.047 ^†,^*
Median (IR)	14.4 (7.4)	14.7 (8.4)	13.9 (7.0)	
Energy intake (Kcal/day)				
Mean + SD	1980.5 ± 519.5	2123.4 ± 548.8	1867.7 ± 466.8	*p* < 0.001 ^‡,^*
(95% CI)	(1918.5–2042.6)	(2024.2–2222.6)	(1792.9–1942.5)	
Median (IR)	1944.2 (643.5)	2067.6 (729.3)	1829.9 (652.0)	
**UCA (TUNISIA)**				
	**Total Sample (*n* = 132)**	**Men (*n* = 46)**	**Women (*n* = 86)**	***p***
Population (%)	100	34.84	65.15	
BMI (Kg/m^2^)				*p* = 0.001 ^†,^*
Mean + SD (95% CI)	21.4 ± 2.8 (20.9–21.8)	22.3 ± 2.8 (21.5–23.2)	20.8 ± 2.7 (20.2–21.4)	
Median (IR)	21.1 (3.6)	22.2 (3.7)	20.6 (4.0)	
BMI-categories (%)				
Underweight	15.9	6.5	20.9	*p* = 0.059 ^§§^
Normal range	75.0	78.3	73.2	
Overweight	7.6	13.0	4.7	
Obese	1.5	2.2	1.2	
BMI-2-categories (%)				*p* = 0.073 ^§^
Underw. + normal rg.	90.9	84.8	94.1	
Overweight + obese	9.1	15.2	5.9	
Total fiber intake (g)				
Mean + SD (95% CI)	15.0 ± 4.8 (14.1–15.8)	14.6 ± 5.6 (13.0–16.3)	15.1 ± 4.3 (14.2–16.1)	*p* = 0.352 ^†^
Median (IR)	14.5 (5.4)	14.0 (5.7)	14.8 (5.4)	
Energy intake (Kcal/day)				
Mean + SD	1843.4 ± 563.1	1841.2 ± 670.5	1844.5 ± 500.6	*p* = 0.567 ^†^
(95% CI)	(1746.4–1940.3)	(1642.1–2040.4)	(1737.2–1951.9)	
Median (IR)	1769.5 (667.0)	1672.5 (786.0)	1801.0 (646.3)	
**FIU (USA)**				
	**Total Sample (*n* = 326)**	**Men (*n* = 73)**	**Women (*n* = 253)**	***p***
Population (%)	100	22.39	77.61	-
BMI (Kg/m^2^)				*p* < 0.001 ^†,^*
Mean + SD (95% CI)	23.9 ± 4.8 (23.4–24.4)	26.2 ± 4.9 (25.1–27.4)	23.2 ± 4.6 (22.6–23.8)	
Median (IR)	23.1 (5.1)	24.9 (5.9)	22.5 (4.9)	
BMI-categories (%)				
Underweight	4.9	1.4	5.9	*p* = 0.001 ^§§,^*
Normal range	64.4	50.7	68.4	
Overweight	20.2	26.0	18.6	
Obese	10.4	21.9	7.1	
BMI-2-categories (%)				*p* < 0.001 ^§,^*
Underw. + normal rg.	69.3	52.1	74.3	
Overweight + obese	30.6	47.9	25.7	
Total fiber intake (g)				
Mean + SD (95% CI)	20.8 ± 12.0 (17.5–22.1)	20.2 ± 11.6 (17.5–22.9)	20.9 ± 12.1 (19.4–22.4)	*p* = 0.393 ^†^
Median (IR)	18.1 (12.8)	17.3 (14.8)	18.1 (12.5)	
Energy intake (Kcal/day)				
Mean + SD	2016.8 ± 596.6	2127.0 ± 623.8	1985.0 ± 550.2	*p* = 0.060 ^‡^
(95% CI)	(1954.7–2078.9)	(1981.5–2272.6)	(1916.9–2053.1)	
Median (IR)	1993.0 (841.5)	2211.0 (947.5)	1947.0 (808.0)	

UCLM: University of Castilla-La Mancha; UCA: University of Carthage; FIU: Florida International University; SD: Standard deviation; CI: Confidence interval; IR: Interquartile range; BMI: Body mass index. ^†^ Mann-Whitney U test; ^‡^ Student’s *t* test; ^§^ *χ*^2^ test; ^§§^ Likelihood ratio test; * Significant differences.

**Table 3:** Sociodemographic, anthropometric, and lifestyle differences among the three populations.

	UCLM (SPAIN)				UCA (TUNISIA)				FIU (USA)				*p*
	Mean + SD	CI 95%	Median	IR	Mean + SD	CI 95%	Median	IR	Mean + SD	CI 95%	Median	IR	
Age (years)	20.3 ± 2.4	20.0–20.5	19.0 ^a^	2	19.8 ± 1.4	19.6–20.1	19.0 ^a^	1	22.6 ± 2.8	22.3–22.9	22.0 ^a,b^	3	*p* < 0.001 ^†,^*
Weight (Kg)	65.7 ± 12.9	64.2–67.3	64	18.0	64.3 ± 11.3	62.4–66.3	63.0	14.0	64.6 ± 14.3	63.0–66.2	61.5	18.0	*p* = 0.278 ^†^
BMI (Kg/m^2^)	22.4 ± 3.3	22.0–22.8	22 ^c,d^	4.1	21.4 ± 2.8	20.9–21.8	21.1 ^c,e^	3.6	23.9 ± 4.8	23.4–24.4	23.1 ^d,e^	5.1	*p* < 0.001 ^†,^*
Total fiber intake (g)	15.7 ± 6.6	14.9–16.5	14.4 ^f^	7.4	15.0 ± 4.8	14.1–15.8	14.5 ^g^	5.4	20.8 ± 12.0	19.5–22.1	18.1 ^f,g^	12.8	*p* < 0.001 ^†,^*
Fiber intake (g/1000 Kcal)	8.1 ± 3.2	7.7–8.5	7.5 ^h^	3.4	8.4 ± 2.2	8.0–8.7	8.0 ^i^	2.7	10.5 ± 5.6	9.9–11.1	8.9 ^h,i^	5.9	*p* < 0.001 ^†,^*
Energy (Kcal/day)	1980.5 ± 519.5	1918.5–2042.6	1944.2 ^j^	643.5	1843.4 ± 563.1	1746.4–1940.3	1769.5 ^j,k^	667.0	2016.8 ± 596.6	1954.7–2078.9	1993.0 ^k^	841.5	*p* = 0.002 ^†,^*
Weight-loss diet (%)													*p* < 0.001 ^§,^*
Yes	5.1				12.1				22.1				
No	94.9				87.9				77.9				
Smoking habits (%)													*p* < 0.001 ^§§,^*
Non-smoker	84.6				77.3				95.4				
≤5 cigarettes/day	8.5				8.3				3.7				
>5 cigarettes/day	7.0				14.4				0.9				
Level of physical activity (%)													*p* < 0.001 ^§§,^*
Sedentary	22.1				1.5				1.2				
Low active	47.4				26.5				12.3				
Active	20.2				43.2				55.8				
Very active	10.3				28.8				30.7				
BMI-categories (%)													*p* < 0.001^§§,^*
Underweight	8.5				15.9				4.9				
Normal range	73.2				75.0				64.4				
Overweight	14.6				7.6				20.2				
Obese	3.7				1.5				10.4				
BMI-2-categories (%)													p < 0.001 §,*
Underw + normal	81.7				90.9				69.3				
Overw. + obese	18.3				9.1				30.6				

UCLM: University of Castilla-La Mancha; UCA: University of Carthage; FIU: Florida International University; SD: Standard deviation; CI: Confidence interval; IR: Interquartile range; BMI: Body mass index. ^†^ Kruskal-Wallis test; ^§^ *χ*^2^ test; ^§§^ Likelihood ratio test; * Significant differences. (1) – (5) Post hoc Dunn’s test. (a) – (k) Data with the same superscript were significantly different.

**Table 4:** Daily total fiber intake (g) by universities for variables presenting significant comparisons.

**UCLM (SPAIN)**						
	***n***	**Mean + SD**	**95% CI**	**Median**	**IR**	***p***
Gender						0.047 ^†,^*
Men	120	16.4 ± 6.3	15.3–17.5	14.7	8.4	
Women	152	15.1 ± 6.9	14.0–16.2	13.9	7.0	
Weight-loss diet						0.691 ^†^
Yes	14	15.3 ± 6.6	11.5–19.1	14.4	10.3	
No	258	15.7 ± 6.6	14.9–16.5	14.4	7.3	
BMI-categories						*p*-trend 0.051 ^§^
Underweight	23	14.2 ± 5.6	11.8–16.7	12.7	5.2	
Normal range	199	16.1 ± 6.8	15.1–17.0	14.8	7.5	
Overweight	40	14.8 ± 6.3	12.8–16.8	13.2	8.3	
Obese	10	14.5 ± 5.8	10.3–18.7	12.1	8.7	
BMI-2-categories						0.190 ^†^
Underw. + normal rg.	222	15.9 ± 6.7	15.0–16.8	14.5	7.2	
Overweight + obese	50	14.7 ± 6.1	13.0–16.5	12.9	8.3	
BMI	*r* = −0.019 (Spearman’s correlation coefficient)					0.753
Energy intake	*r* = 0.462 (Spearman’s correlation coefficient)					<0.001 **
**UCA (TUNISIA)**						
	***n***	**Mean + SD**	**95% CI**	**Median**	**IR**	***p***
Gender						0.352 ^†^
Men	46	14.6 ± 5.6	13.0–16.3	14.0	5.7	
Women	86	15.1 ± 4.3	14.2–16.1	14.8	5.4	
Weight-loss diet						0.734 ^‡^
Yes	16	14.6 ± 6.2	11.3–17.9	14.7	7.1	
No	116	15.0 ± 4.6	14.2–15.9	14.5	5.3	
BMI-categories						*p*-trend 0.471 ^§^
Underweight	21	15.3 ± 4.7	13.2–17.5	13.6	6.4	
Normal range	99	15.0 ± 4.6	14.1–15.9	14.9	5.3	
Overweight	10	14.1 ± 6.7	9.3–18.9	13.2	8.0	
Obese	2	12.1 ± 2.4	0.0–33.7	12.1	-	
BMI-2-categories						0.128 ^†^
Underw. + normal rg.	120	15.1 ± 4.6	14.3–15.9	14.8	5.3	
Overweight + obese	12	13.7 ± 6.2	9.8–17.7	13.0	5.8	
BMI	*r* = −0.053 (Spearman’s correlation coefficient)					0.543
Energy intake	*r* = 0.595 (Spearman’s correlation coefficient)					<0.001 **
**FIU (USA)**						
	***n***	**Mean + SD**	**95% CI**	**Median**	**IR**	***p***
Gender						0.393 ^†^
Men	73	20.2 ± 11.6	17.5–22.9	17.3	14.8	
Women	253	20.9 ± 12.1	19.4–22.4	18.1	12.5	
Weight-loss diet						<0.001 ^†,^*
Yes	72	26.8 ± 15.6	23.2–30.5	22.7	17.7	
No	254	19.0 ± 10.2	17.8–20.3	16.7	11.3	
BMI-categories						*p*-trend 0.322 ^§^
Underweight	16	25.6 ± 18.4	15.7–35.4	20.2	14.2	
Normal range	210	21.0 ± 11.7	19.4–22.6	18.1	13.4	
Overweight	66	19.6 ± 12.7	16.5–22.7	15.3	13.0	
Obese	34	19.3 ± 8.5	16.4–22.3	18.3	8.7	
BMI-2-categories						0.087 ^†^
Underw. + normal rg.	226	21.3 ± 12.3	19.7–22.9	18.5	13.3	
Overweight + obese	100	19.5 ± 11.4	17.2–21.8	15.7	11.0	
BMI	*r* = −0.127 (Spearman’s correlation coefficient)					0.022 **^,(1)^
Energy intake	*r* = 0.449 (Spearman’s correlation coefficient)					<0.001 **

UCLM: University of Castilla-La Mancha; UCA: University of Carthage; FIU: Florida International University; SD: Standard deviation; CI: Confidence interval; IR: Interquartile range; BMI: Body mass index. ^†^ Mann-Whitney U test; ^‡^ Student’s *t* test; ^§^ Jonckheere-Terpstra trend test; * Significant differences; ** Significant correlation; ^(1)^ Correlations by sex: Men: *r* = 0.098, *p* = 0.408; Women: *r* = −0.178, *p* = 0.004 **.

**Table 5:** Fiber intakes (g/1000 Kcal) from the main food groups providing fiber ^§^.

		UCLM (SPAIN)	UCA (TUNISIA)	FIU (USA)	*p* ^†^
Total sample	Cereals	3.3 ± 1.3 (3.1–3.4)	3.6 ± 1.1 (3.4–3.8)	3.0 ± 1.5 (2.9–3.2)	*p* < 0.001 *
		3.0 (1.4) ^a^	3.6 (1.5) ^a,b^	2.9 (2.1) ^b^	
	Legumes	0.5 ± 1.1 (0.4–0.6)	0.5 ± 1.1 (0.3–0.6)	1.2 ± 2.5 (0.9–1.4)	*p* = 0.001 *
		0.000 (0.3) ^c^	0.000 (0.4)	0.000 (1.1) ^c^	
	Vegetables	1.5 ± 1.3 (1.4–1.7)	1.5 ± 1.0 (1.4–1.7)	1.7 ± 1.6 (1.6–1.9)	*p* = 0.360
		1.2 (1.6)	1.4 (1.4)	1.3 (1.7)	
	Fruits	1.1 ± 1.4 (0.9–1.3)	1.3 ± 1.2 (1.0–1.5)	2.0 ± 2.4 (1.7–2.2)	*p* < 0.001 *
		0.6 (1.7) ^d^	1.0 (1.6) ^e^	1.3 (2.2) ^d,e^	
	Total intake	8.1 ± 3.2 (7.7–8.5)	8.4 ± 2.2 (8.0–8.7)	10.5 ± 5.7 (9.9–11.1)	*p* < 0.001 *
		7.5 (3.4) ^f^	8.0 (2.7) ^g^	8.9 (5.9) ^f,g^	
0–9.29 g	Cereals	3.2 ± 1.1 (3.0–3.3)	3.5 ± 1.1 (3.3–3.7)	3.0 ± 1.3 (2.8–3.1)	*p* < 0.001 *
		3.0 (1.4) ^h^	3.5 (1.5) ^h,i^	2.9 (1.8) ^i^	
	Legumes	0.2 ± 0.7 (0.2–0.3)	0.2 ± 0.4 (0.1–0.3)	0.2 ± 0.5 (0.2–0.3)	*p* = 0.224
		0.000 (0.00)	0.000 (0.00)	0.000 (0.04)	
	Vegetables	1.2 ± 1.0 (1.1–1.4)	1.3 ± 0.8 (1.1–1.5)	1.2 ± 0.9 (1.1–1.4)	*p* = 0.670
		1.1 (1.3)	1.2 (1.3)	1.1 (1.2)	
	Fruits	0.8 ± 1.0 (0.6–0.9)	1.1 ± 1.0 (0.9–1.3)	0.9 ± 0.9 (0.8–1.1)	*p* = 0.004 *
		0.5 (1.1) ^j,k^	0.9 (1.4) ^j^	0.7 (1.3) ^k^	
	Total intake	6.7 ± 1.5 (6.5–6.9)	7.3 ± 1.3 (7.0–7.5)	6.7 ± 1.5 (6.5–6.9)	*p* = 0.003 *
		6.7 (2.4) ^l^	7.4 (1.9) ^l,m^	6.8 (2.1) ^m^	
9.30–13.99 g	Cereals	3.7 ± 1.8 (3.3–4.2)	3.9 ± 1.1 (3.5–4.3)	3.1 ± 1.6 (2.8–3.4)	*p* = 0.007 *
		3.4 (1.7)	3.9 (1.7) ^n^	3.0 (2.3) ^n^	
	Legumes	1.0 ± 1.4 (0.6–1.3)	1.2 ± 1.8 (0.6–1.8)	1.2 ± 1.8 (0.8–1.5)	*p* = 0.867
		0.5 (1.7)	0.5 (1.5)	0.000 (1.6)	
	Vegetables	2.3 ± 1.7 (1.9–2.7)	2.2 ± 1.1 (1.8–2.6)	1.9 ± 1.5 (1.6–2.2)	*p* = 0.057
		2.0 (1.4)	2.1 (1.9)	1.5 (1.9)	
	Fruits	2.0 ± 1.8 (1.5–2.4)	1.6 ± 1.4 (1.1–2.1)	2.5 ± 1.9 (2.1–2.9)	*p* = 0.027 *
		2.0 (2.7)	1.3 (2.6) ^o^	2.3 (2.9) ^o^	
	Total intake	11.1 ± 1.3 (10.8–11.4)	10.9 ± 1.3 (10.5–11.4)	11.4 ± 1.2 (11.1–11.6)	*p* = 0.134
		10.9 (2.2)	10.5 (2.1)	11.3 (2.0)	
≥14 g	Cereals	3.5 ± 1.8 (2.2–4.8)	4.2 ± 0.2 (2.6–5.7)	3.1 ± 2.1 (2.6–3.6)	*p* = 0.366
		3.5 (2.7)	4.2 (-)	3.1 (2.6)	
	Legumes	2.2 ± 2.7 (0.3–4.1)	0.2 ± 0.3 (-2.5–3.0)	3.7 ± 4.2 (2.6–4.7)	*p* = 0.426
		1.2 (4.4)	0.2 (-)	2.4 (6.5)	
	Vegetables	1.9 ± 1.5 (0.9–3.0)	2.7 ± 0.6 (-3.03–8.5)	2.9 ± 2.2 (2.3–3.4)	*p* = 0.378
		1.5 (1.9)	2.7 (-)	2.5 (2.8)	
	Fruits	3.0 ± 2.4 (1.3–4.8)	5.0 ± 1.0 (-3.6–13.6)	4.1 ± 3.8 (3.1–5.0)	*p* = 0.474
		2.8 (4.2)	5.0 (-)	3.3 (4.0)	
	Total intake	18.2 ± 6.1(13.8–22.5)	14.7 ± 0.8 (7.3–22.2)	19.4 ± 5.6 (18.0–20.8)	*p* = 0.093
		15.5 (4.4)	14.7 (-)	18.0 (5.6)	

UCLM: University of Castilla-La Mancha; UCA: University of Carthage; FIU: Florida International University. ^†^ Kruskal-Wallis and post hoc Dunn. Data with the same superscript were significantly different; * Significant differences; ^§^ All values are mean ± standard deviation (95% confidence interval) and median (interquartile range).

**Table 6:** Distribution of consumption of high-fiber groups by quartiles of total fiber intake (g) ^§^.

**UCLM (SPAIN)**						
	**TOTAL**	**Q1**	**Q2**	**Q3**	**Q4**	***p*-trend ^†^**
Total	15.7 ± 6.6 (14.9–16.5)	9.1 ± 1.6 (8.8–9.5)	12.8 ± 0.9 (12.6–13.0)	16.5 ± 1.4 (16.1–16.8)	24.3 ± 6.7 (22.6–25.9)	<0.001 *
	14.4 (7.4)	9.5 (2.2)	12.9 (1.4)	16.4 (2.0)	22.7 (6.9)	
Fruits	1.8 ± 2.4 (1.6–2.1)	0.8 ± 1.1 (0.6–1.1)	1.4 ± 1.8 (0.9–1.8)	1.8 ± 2.2 (1.2–2.3)	3.4 ± 3.2 (2.6–4.2)	<0.001 *
	0.9 (2.9)	0.2 (1.4)	0.8 (2.1)	0.7 (3.6)	2.5 (5.0)	
Nuts	0.1 ± 0.4 (0.1–0.2)	0.1 ± 0.4 (0.0–0.2)	0.2 ± 0.6 (0.0–0.3)	0.1 ± 0.3 (0.0–0.2)	0.1 ± 0.3 (0.0–0.2)	0.168
	0.0 (0.0)	0.0 (0.0)	0.0 (0.0)	0.0 (0.0)	0.0 (0.0)	
Legumes	0.9 ± 2.2 (0.7–1.2)	0.2 ± 0.7 (0.0–0.3)	0.2 ± 0.8 (0.04–0.4)	0.7 ± 1.4 (0.4–1.1)	2.6 ± 3.5 (1.8–3.5)	<0.001 *
	0.0 (0.8)	0.0 (0.0)	0.0 (0.0)	0.0 (0.0)	1.6 (3.9)	
Vegetables	2.6 ± 1.9 (2.4–2.8)	1.7 ± 1.4 (1.4–2.1)	2.4 ± 1.9 (2.0–2.9)	3.0 ± 2.0 (2.5–3.5)	3.1 ± 2.0 (2.6–3.6)	<0.001 *
	2.2 (2.6)	1.4 (2.2)	2.0 (2.0)	2.5 (3.1)	2.6 (2.9)	
Cereals	6.5 ± 3.2(6.1–6.9)	4.6 ± 1.7 (4.2–5.0)	6.1 ± 2.5 (5.5–6.7)	7.0 ± 2.9 (6.3–7.7)	8.5 ± 4.0 (7.5–9.4)	<0.001 *
	6.0 (3.9)	4.6 (2.1)	6.0 (3.4)	6.4 (4.7)	8.1 (5.7)	
**UCA (TUNISIA)**						
	**TOTAL**	**Q1**	**Q2**	**Q3**	**Q4**	***p*-trend ^†^**
Total	15.0 ± 4.8 (14.1–15.8)	9.5 ± 1.8 (8.9–10.2)	13.3 ± 0.8 (13.0–13.5)	15.9 ± 0.9 (15.6–16.2)	21.1 ± 3.8 (19.8–22.5)	<0.001 *
	14.5 (5.4)	9.5 (2.3)	13.5 (1.5)	15.7 (1.8)	20.1 (4.6)	
Fruits	2.0 ± 2.1 (1.7–2.4)	0.7 ± 1.1 (0.3–1.1)	1.9 ± 1.6 (1.3–2.4)	2.1 ± 1.9 (1.4–2.8)	3.5 ± 2.5 (2.6–4.4)	<0.001 *
	1.6 (3.0)	0.000 (1.5)	1.6 (2.7)	1.7 (2.4)	2.8 (2.9)	
Nuts	0.2 ± 0.8 (0.0–0.3)	0.04 ± 0.2 (0.0–0.1)	0.2 ± 0.6 (0.0–0.4)	0.1 ± 0.4 (0.0–0.2)	0.5 ± 1.5 (0.0–1.0)	0.351
	0.0 (0.0)	0.0 (0.0)	0.0 (0.0)	0.0 (0.0)	0.0 (0.0)	
Legumes	0.5 ± 1.1 (0.3–0.7)	0.1 ± 0.5 (0.0–0.3)	0.6 ± 1.0 (0.2–0.9)	0.3 ± 0.7 (0.1–0.6)	1.0 ± 1.7 (0.4–1.6)	0.007 *
	0.0 (0.7)	0.0 (0.0)	0.0 (0.7)	0.0 (0.3)	0.0 (1.6)	
Vegetables	2.2 ± 1.5 (20.–2.5)	1.5 ± 1.1 (1.1–1.8)	2.0 ± 1.3 (1.6–2.5)	2.2 ± 1.3 (1.7–2.7)	3.3 ± 1.6 (2.7–3.9)	<0.001 *
	1.9 (2.2)	1.2 (1.2)	1.9 (2.0)	1.8 (2.0)	3.2 (2.5)	
Cereals	5.9 ± 2.2 (5.5–6.3)	4.9 ± 2.0 (4.2–5.6)	5.2 ± 1.9 (4.6–5.9)	6.8 ± 2.3 (5.9–7.6)	6.8 ± 2.0 (6.1–7.5)	<0.001 *
	6.0 (3.0)	4.6 (3.4)	5.4 (3.0)	6.0 (3.2)	6.8 (2.8)	
**FIU (USA)**						
	**TOTAL**	**Q1**	**Q2**	**Q3**	**Q4**	***p*-trend ^†^**
Total	20.8 ± 12.0 (19.5–22.1)	10.0 ± 2.2 (9.5–10.5)	15.3 ± 1.5 (15.0–15.6)	21.1 ± 2.3 (20.6–21.6)	36.8 ± 12.8 (34.0–39.6)	<0.001 *
	18.1 (12.8)	10.4 (2.9)	15.2 (2.3)	20.5 (4.3)	32.9 (11.2)	
Fruits	2.7 ± 3.0 (2.4–3.0)	1.0 ± 1.2 (0.7–1.3)	2.1 ± 1.8 (1.7–2.5)	3.4 ± 3.0 (2.7–4.0)	4.3 ± 4.0 (3.4–5.2)	<0.001 *
	1.8 (3.0)	0.7 (1.6)	1.6 (2.2)	2.5 (3.2)	3.6 (4.8)	
Nuts	0.8 ± 1.4 (0.3–0.6)	0.05 ± 0.3 (0–0.1)	0.3 ± 0.9 (0.1–0.5)	0.6 ± 1.6 (0.2–0.9)	1.0 ± 2.0 (0.5–1.4)	<0.001 *
	0.0 (0.0)	0.0 (0.0)	0.0 (0.0)	0.0 (0.0)	0.0 (0.9)	
Legumes	2.1 ± 4.6 (1.6–2.6)	0.3 ± 0.7 (0.2–0.5)	0.6 ± 1.0 (0.3–0.8)	1.3 ± 2.6 (0.7–1.9)	6.3 ± 7.3 (4.7–7.9)	<0.001 *
	0.000 (1.8)	0.0 (0.0)	0.0 (1.1)	0.0 (1.8)	3.8 (12.4)	
Vegetables	2.5 ± 2.1(2.3–2.8)	1.7 ± 1.4 (1.4–2.00)	2.6 ± 2.1 (2.1–3.1)	2.9 ± 1.9 (2.4–3.3)	3.1 ± 2.6 (2.5–3.6)	<0.001 *
	2.0 (2.7)	1.5 (1.5)	2.1 (2.8)	2.7 (3.1)	2.8 (3.3)	
Cereals	5.8 ± 3.1 (5.5–6.2)	4.6 ± 2.0 (4.2–5.1)	5.6 ± 2.9 (4.9–6.2)	6.8 ± 3.0 (6.1–7.4)	6.4 ± 3.7 (5.6–7.2)	<0.001 *
	5.3 (4.0)	4.9 (2.6)	5.1 (3.6)	6.6 (4.9)	6.5 (5.0)	

UCLM: University of Castilla-La Mancha; UCA: University of Carthage; FIU: Florida International University; ^†^ Jonckheere-Terpstra trend test; * Significant differences; ^§^ All values are mean ± standard deviation (95% confidence interval) and median (interquartile range).

## Supplementary Materials

The following are available online at www.mdpi.com/2072-6643/9/9/1030/s1, Tables S1–S3: Sociodemographic and lifestyle characteristics of the sample by countries (Spain, Tunisia, and the USA), Tables S4–S6: Total fiber intake (g) by countries (Spain, Tunisia, and the USA), and Table S7: Appetizers, prepared and precooked meals, and sauces and condiments consumed by the studied population.
