# Supplementary material for: Fiber Patterns in Young Adults Living in Different Environments (USA, Spain, and Tunisia). Anthropometric and Lifestyle Characteristics
Source: Nutrients. 2017 Sep 18;9(9):1030. doi: 10.3390/nu9091030 (PMC5622790; doi:10.3390/nu9091030)
Supplement: Supplementary file 1 [file nutrients-09-01030-s001.pdf]

Supplementary: Fiber Patterns in Young Adults Living in Different Environments (USA, Spain, and Tunisia). Anthropometric and Lifestyle Characteristics

María José García-Meseguer [1,*], Amalia Delicado-Soria [1] and Ramón Serrano-Urrea [2]

Table S1. Sociodemographic and lifestyle characteristics of the sample by universities (UCLM).

UCLM (SPAIN)				
	Total sample (n = 272)	Men (n = 120)	Women (n = 152)	p
**Population (%)**	100	44.12	55.88	-
**Age (years)**				p = 0.289 †
**Mean + SD (95% CI)**	20.3 ± 2.4 (20.0–20.5)	20.6 ± 2.7 (20.1–21.1)	20.0 ± 2.2 (19.7–20.4)	
**Median (IR)**	19 (2)	19 (3)	19 (2)	
**Weight (Kg)**				P < 0.001†,*
**Mean + SD (95% CI)**	65.7 ± 12.9 (64.2–67.3)	75.2 ± 11.9 (73.0–77.3)	58.3 ± 7.9 (57.0–59.6)	
**Median (IR)**	64 (18.0)	73.3 (15.4)	57.5 (11.9)	
**BMI (Kg/m²)**				p=0.041†,*
**Mean + SD (95% CI)**	22.4 ± 3.3 (22.0–22.8)	22.8 ± 3.4 (22.2–23.4)	22.1 ± 3.2 (21.5–22.6)	
**Median (IR)**	22.0 (4.1)	22.7 (4.2)	21.6 (3.8)	
**BMI-categories (%)**				
**Underweight**	8.5	10	7.2	P = 0.297 §§
**Normal range**	73.2	67.5	77.7	
**Overweight**	14.7	17.5	12.5	
**Obese**	3.7	5.0	2.6	
**BMI-2-categories (%)**				
**Underw. + normal rg.**	81.7	77.5	84.9	p=0.119 §
**Overweight + obese**	18.4	22.5	15.1	
**Total fiber intake (g)**				
**Mean + SD (95% CI)**	15.7 ± 6.6 (14.9–16.5)	16.4 ± 6.3 (15.3–17.5)	15.1 ± 6.9 (14.0–16.2)	p = 0.047 †,*
**Median (IR)**	14.4 (7.4)	14.7 (8.4)	13.9 (7.0)	
**Energy intake (Kcal/day)**				
**Mean + SD**	1980.5 ± 519.5	2123.4 ± 548.8	1867.7 ± 466.8	p < 0.001‡,*
**(95% CI)**	(1918.5–2042.6)	(2024.2–2222.6)	(1792.9–1942.5)	
**Median (IR)**	1944.2 (643.5)	2067.6 (729.3)	1829.9 (652.0)	
**Weight-loss diet (%)**	5.1%	6.7%	3.9%	p=0.715 §
**Smoking habits (%)**				
**Non-smoker**	84.6	83.3	85.5	p=0.420§
**≤5 cigarettes per day**	8.5	7.5	9.2	
**>5 cigarettes per day**	7.0	9.2	5.3	
**Level of physical activity (%)**				
**Sedentary**	22.1	15	27.6	p = 0.001 §,*
**Low active**	47.4	45.8	48.7	
**Active**	20.2	21.7	19.1	
**Very active**	10.3	17.5	4.6	

Abbreviations: UCLM: University of Castilla-La Mancha; SD: standard deviation; CI: Confidence interval; IR: Interquartile range; BMI: body mass index. † Mann-Whitney U test; ‡ Student's t test; § χ^2 test; §§ Likehood ratio test; * Significant differences.

Table S2. Sociodemographic and lifestyle characteristics of the sample by universities (UCA).

**UCA (TUNISIA)**				
	**Total sample (*n* = 132)**	**Men (*n* = 46)**	**Women (*n* = 86)**	***p***
**Population (%)**	100	34.84	65.15	
**Age (years)**				p = 0.001 †,*
**Mean + SD (95% CI)**	19.8±1.4 (29.6-20.1)	20.2±1.4 (19.8-20.6)	19.6±1.4 (19.3-19.9)	
**Median (IR)**	19.0 (1)	20 (2)	19 (1)	
**Weight (Kg)**				p<0.001 †*
**Mean + SD (95% CI)**	64.3±11.3 (62.4-66.3)	72.4±11.1 (69.1-75.7)	60.0±8.8 (58.1-61.9)	
**Median (IR)**	63.0 (14.0)	70.5 (14.3)	59.0 (13.0)	
**BMI (Kg/m^2)**				p=0.001 †*
**Mean + SD (95% CI)**	21.4±2.8 (20.9-21.8)	22.3±2.8 (21.5-23.2)	20.8±2.7 (20.2-21.4)	
**Median (IR)**	21.1 (3.6)	22.2 (3.7)	20.6 (4.0)	
**BMI-categories (%)**				
**Underweight**	15.9	6.5	20.9	p=0.059 §§
**Normal range**	75.0	78.3	73.2	
**Overweight**	7.6	13.0	4.7	
**Obese**	1.5	2.2	1.2	
**BMI-2-categories (%)**				
**Underw. + normal rg.**	90.9	84.8	94.1	p=0.073 §
**Overweight + obese**	9.1	15.2	5.9	
**Total fiber intake (g)**				
**Mean + SD (95% CI)**	15.0±4.8 (14.1-15.8)	14.6±5.6 (13.0-16.3)	15.1±4.3 (14.2-16.1)	p=0.352 †
**Median (IR)**	14.5 (5.4)	14.0 (5.7)	14.8 (5.4)	
**Energy intake (Kcal/day)**				
**Mean + SD (95% CI)**	1843.4±563.1 (1746.4-1940.3)	1841.2±670.5 (1642.1-2040.4)	1844.5±500.6 (1737.2-1951.9)	p=0.567 †
**Median (IR)**	1769.5 (667.0)	1672.5 (786.0)	1801.0 (646.3)	
**Weight-loss diet (%)**	12.1	13.0	11.6	p=0.812 §
**Smoking habits (%)**				
**Non-smoker**	77.3	69.6	81.4	
**≤5 cigarettes per day**	8.3	13.0	5.8	p=0.239 §
**>5 cigarettes per day**	14.4	17.4	12.8	
**Level of physical activity (%)**				
**Sedentary**	1.5	0	2.3	
**Low active**	26.5	17.4	31.4	p=0.124 §§
**Active**	43.2	45.6	41.9	
**Very active**	28.8	37.0	24.4	

Abbreviations: UCA: University of Carthage; SD: standard deviation; CI: Confidence interval; IR: Interquartile range; BMI: body mass index. † Mann-Whitney U test; § χ^2 test; §§ Likehood ratio test; * Significant differences.

Table S3. Sociodemographic and lifestyle characteristics of the sample by universities (FIU).

**FIU (USA)**				
	**Total sample (n=326)**	**Men (n=73)**	**Women (n=253)**	**p**
Population (%)	100	22.39	77.61	-
Age (years)				
**Mean+SD (95% CI)**	22.6±2.8 (22.3-22.9)	23.0±3.1 (22.3-23.7)	22.5±2.7 (22.2-22.9)	p=0.193†
**Median (IR)**	22.0 (3)	23 (4)	22 (3)	
Weight (Kg)				
**Mean+SD (95% CI)**	64.6±14.3 (63.0-66.2)	78.5±13.2 (75.5-81.6)	60.6±11.9 (59.1-62.0)	p<0.001 †*
**Median (IR)**	61.5 (18.0)	77.0 (18.0)	59.0 (14.0)	
BMI (Kg/m^2)				
**Mean+SD (95% CI)**	23.9±4.8 (23.4-24.4)	26.2±4.9 (25.1-27.4)	23.2±4.6 (22.6-23.8)	p<0.001 †*
**Median (IR)**	23.1 (5.1)	24.9 (5.9)	22.5 (4.9)	
BMI-categories (%)				
**Underweight**	4.9	1.4	5.9	
**Normal range**	64.4	50.7	68.4	p=0.001 §§*
**Overweight**	20.2	26.0	18.6	
**Obese**	10.4	21.9	7.1	
BMI-2-categories (%)				
**Underw. + normal rg.**	69.3	52.1	74.3	p<0.001 §*
**Overweight + obese**	30.6	47.9	25.7	
Total fiber intake (g)				
**Mean+SD (95% CI)**	20.8±12.0 (17.5-22.1)	20.2±11.6 (17.5-22.9)	20.9±12.1 (19.4-22.4)	p=0.393 †
**Median (IR)**	18.1 (12.8)	17.3 (14.8)	18.1 (12.5)	
Energy intake (Kcal/day)				
**Mean+SD (95% CI)**	2016.8±596.6 (1954.7-2078.9)	2127.0±623.8 (1981.5-2272.6)	1985.0±550.2 (1916.9-2053.1)	p=0.060 ‡
**Median (IR)**	1993.0 (841.5)	2211.0 (947.5)	1947.0 (808.0)	
Weight-loss diet (%)	22.1	28.8	20.2	p=0.118 §
Smoking habits (%)				
**Non-smoker**	95.4	93.1	96.0	
**≤5 cigarettes per day**	3.7	5.5	3.2	p=0.610 §§
**>5 cigarettes per day**	0.9	1.4	0.8	
Level of physical activity (%)				
**Sedentary**	1.2	0	1.6	
**Low active**	12.3	6.8	13.8	
**Active**	55.8	42.5	59.7	p<0.001 §§*
**Very active**	30.7	50.7	24.9	

Abbreviations: FIU: Florida International University; SD: standard deviation; CI: Confidence interval; IR: Interquartile range; BMI: body mass index; † Mann-Whitney U test; ‡ Student's t test; § χ^2 test; §§ Likehood ratio test; * Significant differences.

Table S4. Total fiber intake (g) by universities (UCLM)

UCLM (SPAIN)						
	**n**	**Mean+SD**	**95% CI**	**Median**	**IR**	**p**
Sex						
**Men**	120	16.4±6.3	15.3-17.5	14.7	8.4	0.047 †*
**Women**	152	15.1±6.9	14.0-16.2	13.9	7.0	
Weight-loss diet						
**Yes**	14	15.3±6.6	11.5-19.1	14.4	10.3	0.691 †
**No**	258	15.7±6.6	14.9-16.5	14.4	7.3	
Smoking habits						
**Non-smoker**	230	15.9±6.9	15.0-16.8	14.4	7.8	p-trend
**≤5 cigarettes per day**	23	15.1±4.4	13.2-17.0	16.7	6.4	0.570 §
**>5 cigarettes per day**	19	14.0±5.9	11.2-16.8	12.5	9.1	
Level of physical activity						
**Sedentary**	60	14.9±5.9	13.4-16.4	13.1	7.9	
**Low active**	129	16.1±7.5	14.8-17.4	14.5	8.2	p-trend
**Active**	55	14.9±5.2	13.5-16.3	14.4	6.3	0.703 §
**Very active**	28	16.9+6.3	14.4-19.3	15.3	5.8	
BMI-categories						
**Underweight**	23	14.2±5.6	11.8-16.7	12.7	5.2	
**Normal range**	199	16.1±6.8	15.1-17.0	14.8	7.5	p-trend
**Overweight**	40	14.8±6.3	12.8-16.8	13.2	8.3	0.051 §
**Obese**	10	14.5±5.8	10.3-18.7	12.1	8.7	
BMI-2-categories						
**Underw.+ normal rg.**	222	15.9±6.7	15.0-16.8	14.5	7.2	0.190 †
**Overweight + obese**	50	14.7±6.1	13.0-16.5	12.9	8.3	
BMI	r= -0.019 (Spearman's correlation coefficient)					0.753
Age	r= 0.014 (Spearman's correlation coefficient)					0.813
Weight	r=0.034 (Spearman's correlation coefficient)					0.580
Energy intake	r=0.462 (Spearman's correlation coefficient)					<0.001 **

Abbreviations: UCLM: University of Castilla-La Mancha; SD: standard deviation; CI: Confidence interval; IR: Interquartile range; BMI: body mass index. † Mann-Whitney U test; § Jonckheere-Terpstra trend test; * Significant differences; ** Significant correlation.

Table S5. Total fiber intake (g) by universities (UCA).

UCA (TUNISIA)						
	**n**	**Mean + SD**	**95% CI**	**Median**	**IR**	**p**
Sex						
**Men**	46	14.6±5.6	13.0-16.3	14.0	5.7	0.352 †
**Women**	86	15.1±4.3	14.2-16.1	14.8	5.4	
Weight-loss diet						
**Yes**	16	14.6±6.2	11.3-17.9	14.7	7.1	0.734 ‡
**No**	116	15.0±4.6	14.2-15.9	14.5	5.3	
Smoking habits						
**Non-smoker**	102	15.0±4.4	14.2-15.9	14.9	5.3	p-trend
**≤5 cigarettes per day**	11	16.2±8.1	10.7-21.7	13.6	8.5	0.290 §
**>5 cigarettes per day**	19	13.9±4.4	11.8-16.0	13.7	8.7	
Level of physical activity						
**Sedentary**	2	14.6±7.8	0.0-87.5	14.6	-	
**Low active**	35	15.7±4.5	14.2-17.8	15.4	5.8	p-trend
**Active**	57	14.5±3.8	13.5-15.5	14.2	4.1	0.765 §
**Very active**	38	15.0±6.1	13.0-17.0	13.7	8.4	
BMI-categories						
**Underweight**	21	15.3±4.7	13.2-17.5	13.6	6.4	
**Normal range**	99	15.0±4.6	14.1-15.9	14.9	5.3	p-Trend
**Overweight**	10	14.1±6.7	9.3-18.9	13.2	8.0	0.471 §
**Obese**	2	12.1±2.4	0.0-33.7	12.1	-	
BMI-2-categories						
**Underw.+ normal rg.**	120	15.1±4.6	14.3-15.9	14.8	5.3	0.128 †
**Overweight + obese**	12	13.7±6.2	9.8-17.7	13.0	5.8	
BMI	r= -0.053 (Spearman's correlation coefficient)					0.543
Age	r=0.147 (Spearman's correlation coefficient)					0.092
Weight	r=0.115 (Spearman's correlation coefficient)					0.189
Energy intake	r=0.595 (Spearman's correlation coefficient)					<0.001 **

Abbreviations: UCA: University of Carthage; SD: standard deviation; CI: Confidence interval; IR: Interquartile range; BMI: body mass index. † Mann-Whitney U test; ‡ Student's t test; § Jonckheere-Terpstra trend test; ** Significant correlation.

Table S6. Total fiber intake (g) by universities (FIU)

	**FIU (USA)**					
	**n**	**Mean+SD**	**95% CI**	**Median**	**IR**	**p**
Sex						
**Men**	73	20.2±11.6	17.5-22.9	17.3	14.8	0.393†
**Women**	253	20.9±12.1	19.4-22.4	18.1	12.5	
Weight-loss diet						
**Yes**	72	26.8±15.6	23.2-30.5	22.7	17.7	<0.001 †*
**No**	254	19.0±10.2	17.8-20.3	16.7	11.3	
Smoking habits						
**Non-smoker**	311	20.7±12.1	19.3-22.0	18.0	12.5	p-trend 0.255 §
**≤5 cigarettes per day**	12	24.1±11.1	17.1-31.2	25.0	19.7	
**>5 cigarettes per day**	3	18.5±11.4	0.0-46.8	20.9	-	
Level of physical activity						
**Sedentary**	4	24.7±28.7	0.0-70.4	11.7	46.4	p-trend 0.034 §
**Low active**	40	18.6±6.9	16.4-20.8	18.2	11.0	
**Active**	182	20.0±12.0	18.3-21.7	16.5	14.0	
**Very active**	100	22.9±12.7	20.4-25.4	19.7	11.8	
BMI-categories						
**Underweight**	16	25.6±18.4	15.7-35.4	20.2	14.2	p-trend 0.322 §
**Normal range**	210	21.0±11.7	19.4-22.6	18.1	13.4	
**Overweight**	66	19.6±12.7	16.5-22.7	15.3	13.0	
**Obese**	34	19.3±8.5	16.4-22.3	18.3	8.7	
BMI-2-categories						
**Underw.+ normal rg.**	226	21.3±12.3	19.7-22.9	18.5	13.3	0.087 †
**Overweight + obese**	100	19.5±11.4	17.2-21.8	15.7	11.0	
BMI	r= -0.127 (Spearman's correlation coefficient)					0.022 ** (1)
Age	r= 0.071 (Spearman's correlation coefficient)					0.202
Weight	r=0.089 (Spearman's correlation coefficient)					0.107
Energy intake	r=0.449 (Spearman's correlation coefficient)					<0.001 **

Abbreviations: FIU: Florida International University; SD: standard deviation; CI: Confidence interval; IR: Interquartile range; BMI: body mass index. † Mann-Whitney U test; § Jonckheere-Terpstra trend test; * Significant differences; ** Significant correlation; (1) Correlations by sex: Men: r=0.098, p=0.408; Women: r=-0.178, p=0.004 **.

Table S7. Appetizers, prepared and precooked meals, and sauces and condiments consumed by the studied population.

SPAIN			
	Appetizers		Saltine crackers; Potato chips; Corn chips; Popcorn; Wheat-based snacks; Corn snacks; Nacho cheese tortilla chips; Cucumber pickles; Black pitted olives, Green pitted olives
	Prepared and precooked meals		Canned meatballs; Frozen fried rice; Squid rings fried in batter; Beef cube bouillon; Frozen meat cannelloni; Frozen cod croquettes; Frozen ham croquettes; Frozen chicken croquettes; Frozen tuna patties; Frozen puff pastry; Frozen vegetable ratatouille; Frozen French fried potatoes; Frozen tuna pizza; Frozen margherita pizza; frozen breaded chicken; Frozen spring rolls; Cheese and ham in breadcrumbs; Canned gazpacho; Poultry noodle soup
	Sauces and condiments	Sauces	Garlic sauce; fried tomato; Soy sauce; Bolognese sauce; Bechamel sauce; Cheese sauce; Barbecue sauce; Mustard; Ketchup
		Condiments	Parsley flakes; Paprika; Ground white pepper; Ground black pepper; Hot chilli pepper; Dried oregano; Ground saffron; Ground nutmeg; Bay leaves; Ground cinnamon
TUNISIA			
	Appetizers		Saltine crackers; Potato chips; Corn chips; Popcorn
	Prepared and precooked meals		Beef cube bouillon; Frozen tuna pizza
	Sauces and condiments	Sauces	Ketchup
		Condiments	Parsley flakes; Paprika; Ground white pepper; Ground black pepper; Dried oregano; Ground saffron; Ground nutmeg; Bay leaves; Ground cinnamon; Fresh mint; Thyme leaves; Ground ginger; Caraway seed; Cardamom; Turmeric; Ground cumin; Fennel seed
USA			

Appetizers		Non-pareil capers; Saltine crackers; Potato chips; Popcorn; Corn snacks; Nacho cheese; Cucumber pickles; Black pitted olives, Green pitted olives; Patacones; Mixed nuts; Pretzel crisps; Hash brown
Prepared and precooked meals		Canned meatballs; Beef cube bouillon; Cream of mushroom soup; Frozen cod croquettes; Frozen ham croquettes; Frozen crunchy breaded fish fillets; Fish patty; Frozen tuna patties; Frozen creamed spinach; Frozen puff pastry; Frozen breaded chicken; Frozen ravioli with tomato sauce; Mashed potatoes; Vegetable soup; Potato salad; Tropichop; Cheese burger; Crispy chicken wrap; Turkey burger; Homewrecker burrito; Beef steak; Sausage and pepperoni; Chicken sandwich with honey oat; Pepperoni salad Chiplotle; Tuna foot long; Quarter pounder deluxe; Cheese sticks; Sesame chicken; Big mac burger; Ramen noodle soup; Turkey and ham; Mac&cheese; Chicken soup; Caesar salad wrap; Chicken strips; Buffalo wings; Seasoned curly fries; Chicken bites; Deluxe breakfast; Grilled sausage; Chicken marsala; Chicken soup; Chicken and bacon sandwich; Macaroni and cheese; Grilled chicken sandwich; Whopper with cheese; Turkey and American cheese
Sauces and condiments	Sauces	Fried tomato; Soy sauce; Bolognese sauce; Cheese sauce; Mustard; Ketchup; Sweet & sour sauce; Alfredo sauce; Barbecue sauce
	Condiments	Parsley flakes; Paprika; Ground white pepper; Ground black pepper; Spicy chilli; Dried oregano; Ground saffron; Ground nutmeg; Bay leaves; Ground cinnamon; Fresh mint; Thyme leaves; Ground ginger; Caraway seed; Ground cumin; Vanilla extract; Ground coriander; Basil leaves
